# A structural variant in the 5’-flanking region of the *TWIST2* gene affects melanocyte development in belted cattle

**DOI:** 10.1371/journal.pone.0180170

**Published:** 2017-06-28

**Authors:** Nivedita Awasthi Mishra, Cord Drögemüller, Vidhya Jagannathan, Irene Keller, Daniel Wüthrich, Rémy Bruggmann, Julia Beck, Ekkehard Schütz, Bertram Brenig, Steffi Demmel, Simon Moser, Heidi Signer-Hasler, Aldona Pieńkowska-Schelling, Claude Schelling, Marcos Sande, Ronald Rongen, Stefan Rieder, Robert N. Kelsh, Nadia Mercader, Tosso Leeb

**Affiliations:** 1 Institute of Genetics, Vetsuisse Faculty, University of Bern, Bern, Switzerland; 2 DermFocus, Vetsuisse Faculty, University of Bern, Bern, Switzerland; 3 Swiss Competence Center of Animal Breeding and Genetics, University of Bern, Bern University of Applied Sciences HAFL & Agroscope, Bern, Switzerland; 4 Department of Clinical Research, University of Bern, Bern, Switzerland; 5 Swiss Institute of Bioinformatics, Bern, Switzerland; 6 Interfaculty Bioinformatics Unit, University of Bern, Bern, Switzerland; 7 Chronix Biomedical, Göttingen, Germany; 8 Institute of Veterinary Medicine, University of Goettingen, Göttingen, Germany; 9 Bern University of Applied Sciences, School of Agricultural, Forest and Food Sciences, Zollikofen, Switzerland; 10 Clinic for Reproductive Medicine, University of Zurich, Zurich, Switzerland; 11 Institute of Anatomy, University of Bern, Bern, Switzerland; 12 Dutch Belted Cattle Association, EM Eefde, The Netherlands; 13 Agroscope, Swiss National Stud Farm, Avenches, Switzerland; 14 Department of Biology and Biochemistry, University of Bath, Claverton Down, Bath, United Kingdom; Stanford University, UNITED STATES

## Abstract

Belted cattle have a circular belt of unpigmented hair and skin around their midsection. The belt is inherited as a monogenic autosomal dominant trait. We mapped the causative variant to a 37 kb segment on bovine chromosome 3. Whole genome sequence data of 2 belted and 130 control cattle yielded only one private genetic variant in the critical interval in the two belted animals. The belt-associated variant was a copy number variant (CNV) involving the quadruplication of a 6 kb non-coding sequence located approximately 16 kb upstream of the *TWIST2* gene. Increased copy numbers at this CNV were strongly associated with the belt phenotype in a cohort of 333 cases and 1322 controls. We hypothesized that the CNV causes aberrant expression of *TWIST2* during neural crest development, which might negatively affect melanoblasts. Functional studies showed that ectopic expression of bovine *TWIST2* in neural crest in transgenic zebrafish led to a decrease in melanocyte numbers. Our results thus implicate an unsuspected involvement of TWIST2 in regulating pigmentation and reveal a non-coding CNV underlying a captivating Mendelian character.

## Introduction

Coat color has been a long-standing model trait for geneticists due to the ease of phenotype recording. Coat color depends on the presence of pigment cells. In mammals, melanin-synthesizing melanocytes, which occupy the skin and hair follicles throughout the entire body surface, are responsible for pigmentation [1]. During embryonic development melanocytes are formed from melanoblasts, which originate in the neural crest and migrate through the developing embryo in order to reach their final position on the body [2]. This developmental program requires a delicate level of regulation to ensure that the correct number of cells reaches their final destination [3–5]. An over-proliferation of cells that have left their surrounding tissue leads to naevi, and in rare cases to congenital melanomas [6–8]. In contrast, if too few melanoblasts are produced or survive, this will lead to partially or completely unpigmented phenotypes, including so-called “white-spotting” phenotypes characterized by patches of unpigmented skin and/or hair [9]. Domestic animals with such hypopigmented phenotypes have been highly valued due to their striking appearance and have often been actively selected in animal breeding. Modern domestic animals thus constitute a unique reservoir of spontaneous pigmentation mutants. Their favorable population structure facilitates the discovery of functional genetic variation including some interesting non-coding structural variants with regulatory effects [10–15].

A symmetrical belt of unpigmented skin circling the midsection has been observed in several mammalian species. It is thought that these belted phenotypes are due to downregulated melanoblast formation or early melanoblast losses in neural crest development. Belted mice have sequence variants in the *Adamts20* gene encoding a secreted metalloprotease [16], which was shown to be required for melanoblast survival [17]. A complex structural rearrangement involving duplication of the *KIT* gene was identified in belted pigs, whose belt includes the forelimbs and is localized more cranially than the one in *Adamts20* mutant mice [18,19].

**Fig 1:**
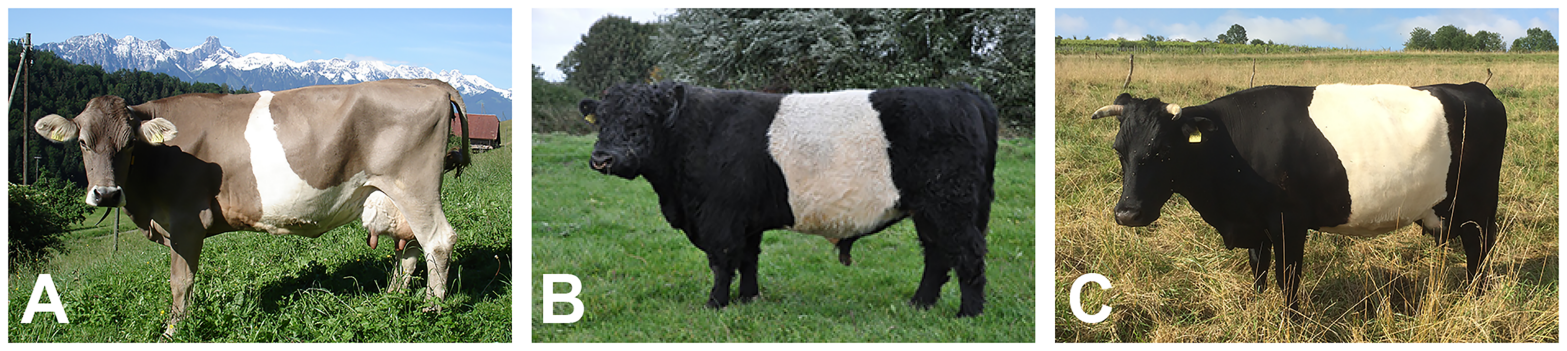
Belted phenotype in different cattle breeds. (A) Brown Swiss. (B) Belted Galloway. (C) Lakenvelder or Dutch Belted. The width and shape of the belt is variable and in some belted animals the unpigmented area does not fully circle the animal.

Belted cattle exist in at least three different breeds: Brown Swiss, (Belted) Galloway, and Lakenvelder, which are also known as Dutch Belted. The belt in these cattle breeds has an intermediate position; it is more caudal than the belt in pigs and more cranial than the belt in *Adamts20* mutant mice (Fig 1). The belt in cattle is inherited as a monogenic autosomal dominant trait and linked to the telomeric end of chromosome 3, which does not contain any known coat color gene ([20,21] OMIA 001469–9913). The objective of this study was to identify the causative genetic variant and to provide a hypothesis on the functional mechanism leading to the partial lack of melanocytes in belted cattle.

## Results

### Positional cloning of the belt sequence variant reveals a CNV upstream of *TWIST2*

**Fig 2:**
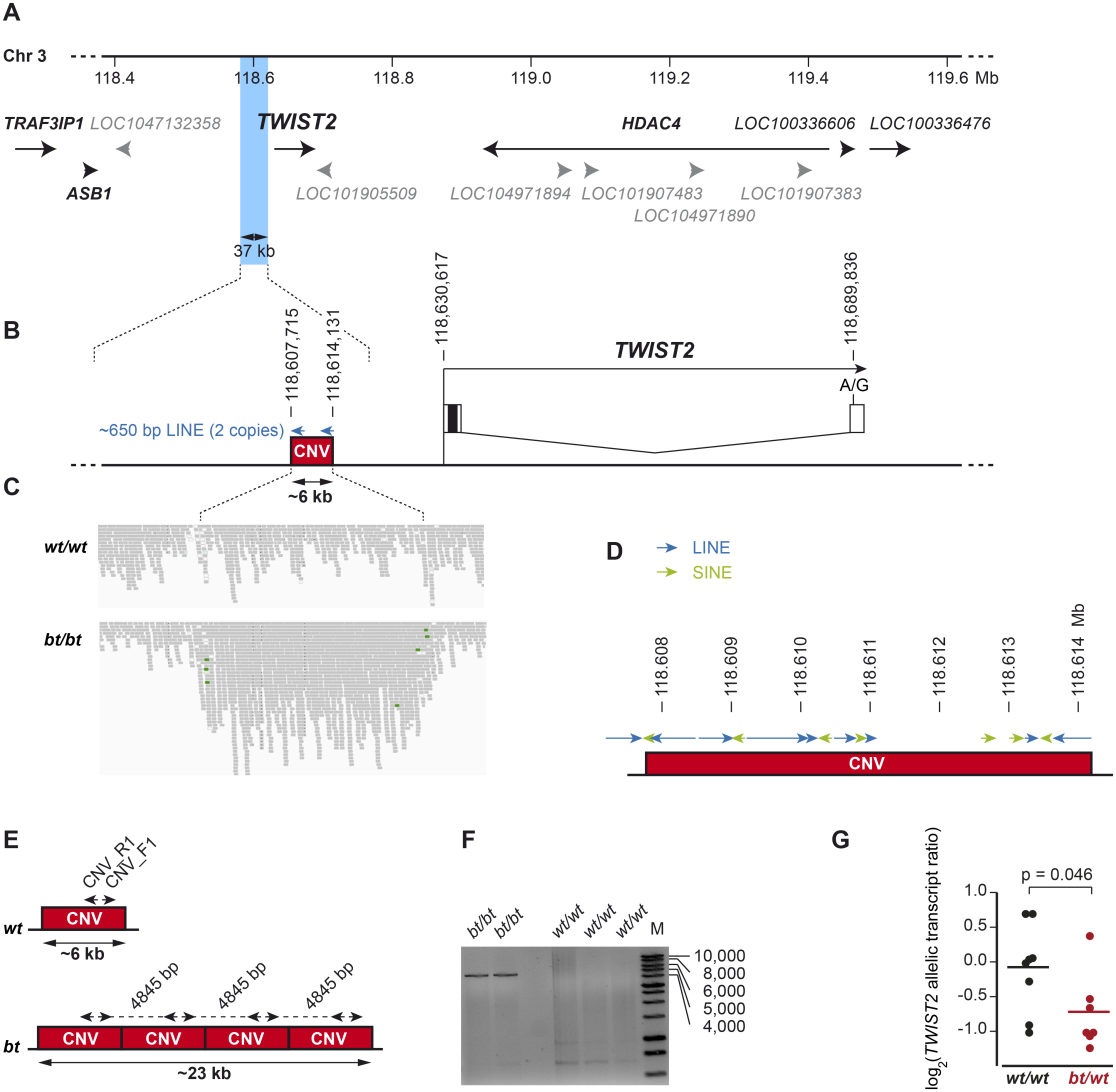
Genomic context of the belt locus. (A) Haplotype analysis defined a critical interval of 37 kb indicated by blue shading for the belt causative variant (Chr3:118,578,893–118,616,348, UMD3.1 assembly, S1 Table). The belt locus mapped to a gene poor region containing *TWIST2* as the only known gene (NCBI annotation release 105; protein coding genes are shown in black, predicted non-coding RNA genes in grey). (B) A 6 kb CNV is located within the critical interval and approximately 16 kb upstream of the transcription start site of *TWIST2*. The CNV is flanked by two highly homologous LINE sequences that share 94% sequence identitiy over 650 bp. (C) Experimental identification of the CNV. IGV screenshots of the illumina short read sequences illustrate a ~4-fold increased coverage in a homozygous belted (*bt/bt*) cattle with respect to a control animal (*wt/wt*) and several read-pairs with incorrect read-pair orientation at the boundaries of the CNV (indicated in green). (D) The CNV is largely composed of interspersed repeats. However, it has a short single copy region, which is highly conserved in mammals. (E) Inverse PCR strategy to confirm the presence of tandemly repeated copies. (F) Agarose gel showing the expected 4845 bp amplicon that is diagnostic for the amplified CNV in belted animals. (G) Allele-specific quantification of *TWIST2* mRNA expression in adult skin. RNA-seq data were analyzed from non-belted (*wt/wt*) and heterozygous belted (*bt/wt*) animals. All animals were heterozygous for an A/G SNV in the 3’-UTR of *TWIST2*. In *wt/wt* animals the two *TWIST2* alleles were expressed at equal amounts. In *bt/wt* animals, the G-allele transcribed from the *bt* haplotype was reduced by 35% compared to the A-allele (p = 0.046, two-sided t-test).

We had previously shown that the belt locus (*bt*) maps to a 336 kb interval at the telomeric end of chromosome 3 and that belted animals from the Brown Swiss, Belted Galloway and Lakenvelder breeds share the same haplotype in the critical interval [21]. In order to further refine this interval, we now genotyped 112 belted cattle on the Illumina bovine high density beadchip, phased the genotype data and searched for a shared haplotype. All belted animals had at least one copy of a shared haplotype spanning 12 consecutive markers (S1 Table). Thus, the refined boundaries of the new critical interval are defined by the flanking markers on either side of this shared haplotype block. The refined critical interval consisted of only 37 kb and spanned from position 118,578,893 to 118,616,348 on chromosome 3 (Fig 2A).

For the identification of potentially causative genetic variants, we sequenced the genomes of two homozygous *bt/bt* cattle and compared the data to 130 genomes from control cattle. A standard bioinformatic pipeline for the detection of single nucleotide variants or small indels did not reveal any private homozygous variants in the two cases in the critical interval. We therefore visually inspected the short-read sequencing data in the critical interval and identified an amplification of a 6 kb sequence region (Fig 2B, 2C and 2D). Both belted animals had a ~4-fold increased read coverage with respect to control animals. The 6 kb CNV was located approximately 16 kb upstream of the *TWIST2* gene. We did not detect any other structural variant in the critical interval.

We designed a digital droplet PCR (ddPCR) assay to determine the copy number of the CNV in a large cohort of 333 belted cattle and 1322 non-belted cattle. The observed copy numbers varied between 2 and 12 with the most frequently observed values being 2, 5 and 8, which most likely corresponded to the copy number genotypes *1/1*, *1/4*, and *4/4*. The belt phenotype was strongly associated with an increased copy number at the CNV (Table 1; S2 Table). The copy number was not associated with the width, position, or regularity of the belt.

**Table 1:** Association of the belt phenotype with the CNV.

Copy number	Belted cattle (N = 333)	Non-belted cattle (N = 1322)
2	2^a^	1322
3	2	-
4	16	-
5	94	-
6	19	-
7	51	-
8	138	-
9	10	-
12	1	-

^a^We observed a nearly perfect association between increased copy number and the belted phenotype. The two discordant animals in this analysis may in fact have been incorrectly phenotyped (see Materials and methods).

### Analysis of the CNV sequence

Several attempts to amplify the entire quadruplicated region by long-range PCR from a homozygous *bt/bt* animal failed. We therefore employed an inverse PCR strategy with outward facing primers to amplify at least parts of the CNV. This experiment indicated that at least two of the four copies must be oriented in tandem orientation (Fig 2E and 2F). The CNV contains ~650 bp partial LINE sequences at its beginning and end. The two sequences share 94% sequence identity and may possibly haven given rise to one or more unequal crossing over events that caused the amplification of this sequence. The CNV is mostly composed of consecutive LINE and SINE elements, which comprise more than 70% of its sequence (Fig 2D; S3 Table). The only single copy regions span from chr3:118,611,095–118,612,598 and from chr3:118,612,871–118,613,048. The single copy regions show high evolutionary conservation within mammals. The orthologous human segment contains a DNaseI hypersensitive cluster, which may indicate a potential regulatory function (human GRCh38.p7 assembly: chr2:238,821,366–238,822,766).

### Does the CNV affect TWIST2 expression?

TWIST2 is normally expressed in the skin and craniofacial cartilages, but not in the neural crest [22,23]. We performed a global RNA-seq experiment to quantify *TWIST2* RNA expression in adult skin from belted and non-belted cattle. We did not detect any significant differences in *TWIST2* expression between belted and non-belted cattle or between the pigmented and non-pigmented skin in belted cattle (S4 Table).

We then performed a second RNA-seq experiment to investigate the allele-specific *TWIST2* expression levels in heterozygous *bt/wt* cattle. For this experiment we selected animals that were heterozygous for an A/G single nucleotide variant in the 3’-UTR of the *TWIST2* gene. In non-belted animals that had the *1/1* genotype at the CNV, both alleles were equally expressed. In contrast, in the skin from heterozygous belted animals with the *1/4* genotype at the CNV, the *bt* allele with 4 copies of the CNV gave rise to only ~65% of the transcript levels compared to the *wt* allele with a single copy of the CNV (Fig 2G).

The strictly dominant mode of inheritance of the belt suggests a gain of function of the *bt* allele. Given that the boundaries of the unpigmented area in belted animals are very even and mostly bilaterally symmetrical, this gain of function might supposedly take place during early melanoblast development. We hypothesized that increased copy numbers at the CNV might lead to an ectopic expression of TWIST2 during neural crest development and thus show the opposite regulatory effect as observed in adult skin. Accordingly, ectopically expressed TWIST2 might decrease the number of melanoblasts and lead to the unpigmented skin area of the belt. Unfortunately, it is currently not possible to test this hypothesis by directly quantifying TWIST2 expression in the developing neural crest of bovine embryos.

### Functional analysis of TWIST2 overexpression

As bovine embryos are not readily accessible for experimental research, we investigated the effect of TWIST2 overexpression in transgenic zebrafish embryos. The TWIST2 protein is highly conserved among vertebrates. Bovine TWIST2 (160 aa) is 100% identical with human TWIST2 and it has 85% identity with zebrafish twist2 (163 aa). We prepared expression constructs with a zebrafish *mitfa* promoter fragment that has been shown to drive expression in premigratory neural crest cells and differentiating melanocytes [24,25]. The control construct pmitfa_EGFP contained the coding sequence of enhanced green fluorescent protein (EGFP) as a reporter under the control of the *mitfa* promoter. Additionally we prepared a second construct pmitfa_btaTWIST2_EGFP, which contained the coding sequence for bovine TWIST2.

**Fig 3:**
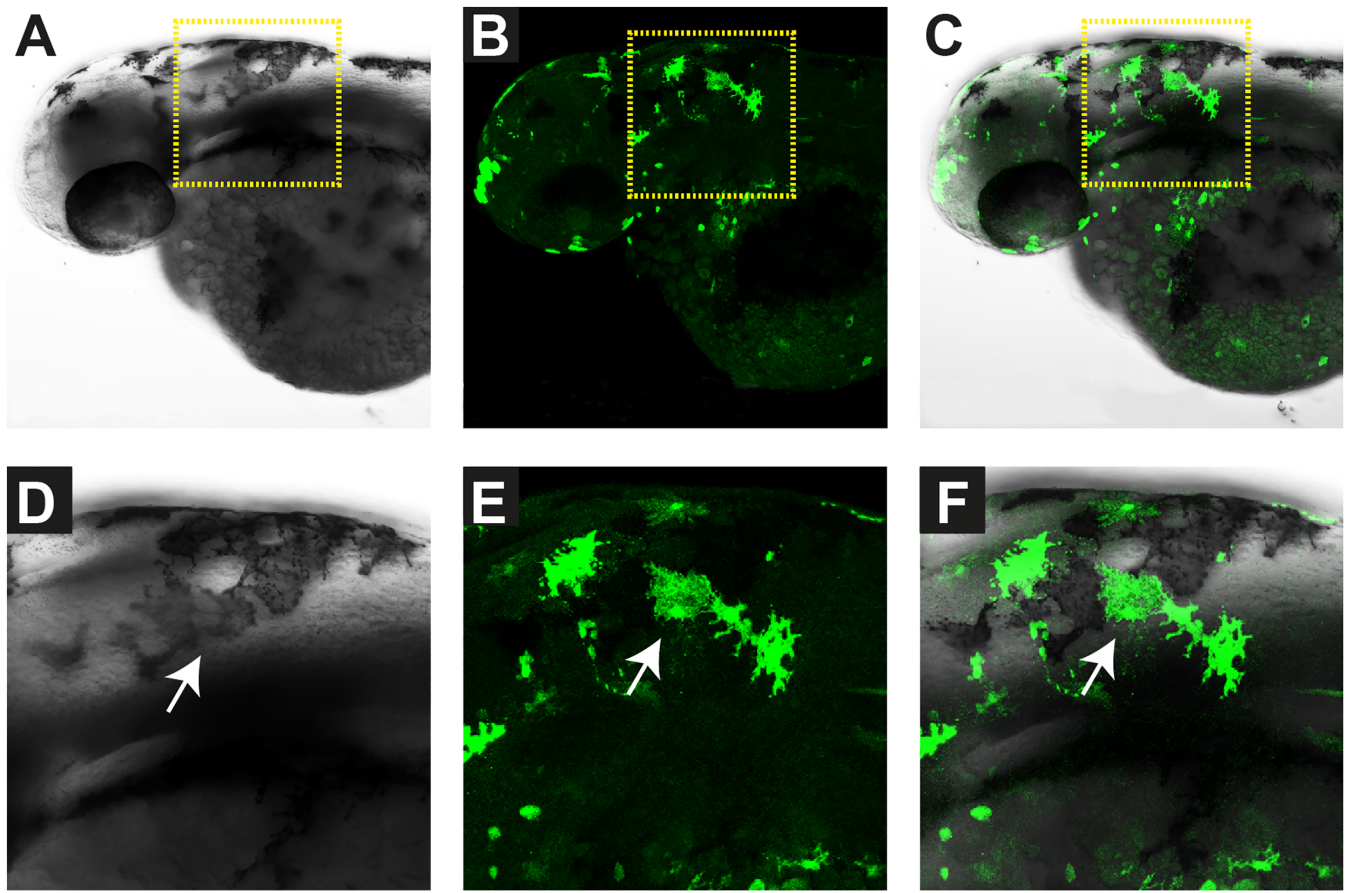
Verification of EGFP expression in melanocytes. In all panels, representative images of the same zebrafish embryo at 35 hours post fertilization (hpf) are shown. This embryo had been injected with the control construct pmitfa_EGFP, which drives expression of green fluorescent protein under the control of the zebrafish mitfa promoter. (A) The brightfield image shows individual darkly pigmented melanocytes. (B) EGFP expression was confirmed by antibody staining with an anti-EGFP antibody resulting in green fluorescence. (C) Superimposition of the brightfield image showing melanocytes with the corresponding fluorescent image showing EGFP positive cells. (D-F) Magnifications of the areas indicated by the dashed yellow square. An individual EGFP-positive melanocyte is indicated by arrows.

We injected pmitfa_EGFP into fertilized zebrafish eggs and analyzed the expression of EGFP at 35 hours post fertilization (hpf) in four replicate experiments. This confirmed the presence of several EGFP-positive cells in a pattern resembling developing melanoblasts (Fig 3). We additionally stained for the expression of sox10 to investigate whether there were any gross changes to neural crest development. The comparison of sox10 expression patterns did not indicate any major differences between the control embryos and the embryos overexpressing TWIST2.

**Fig 4:**
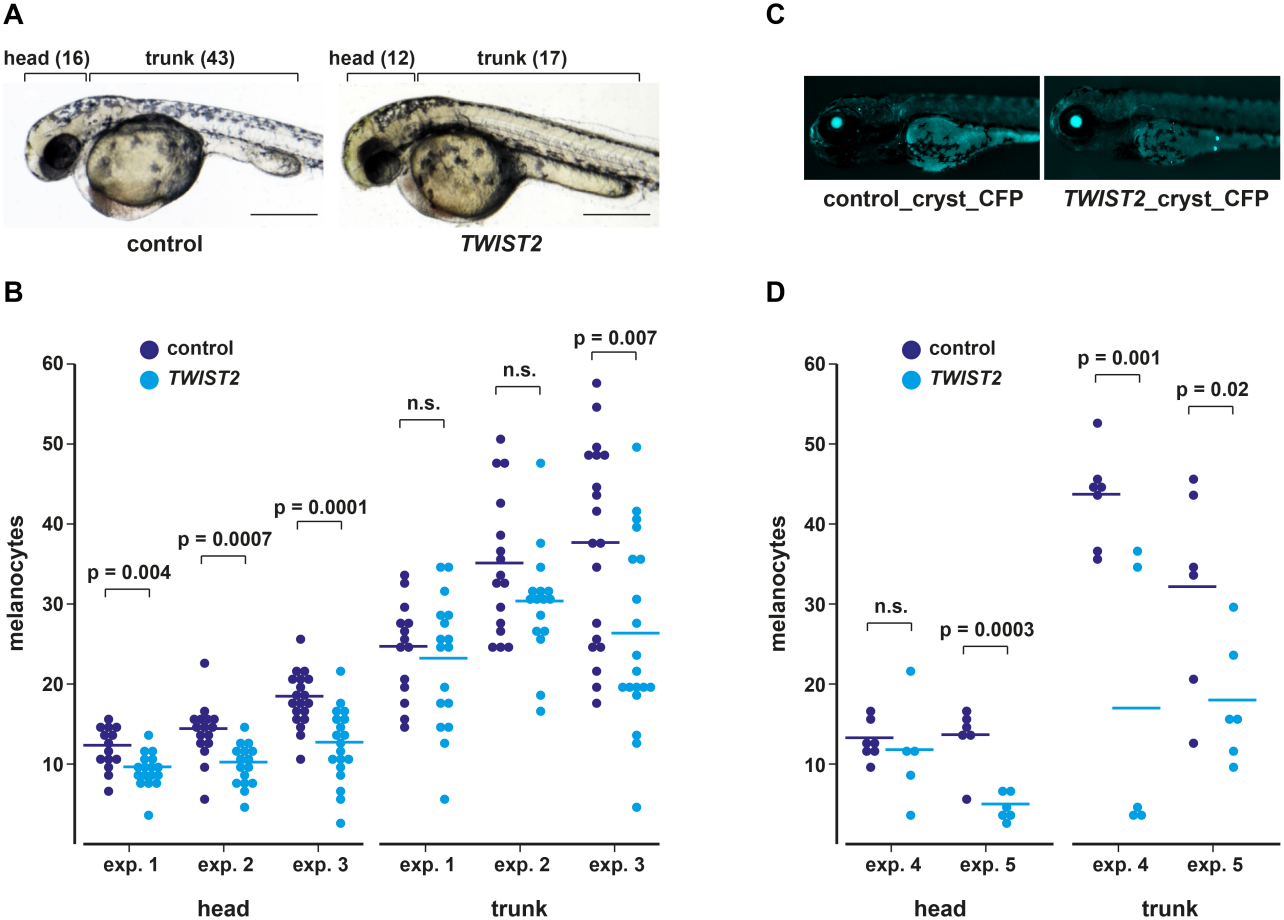
Reduction of melanocytes in zebrafish embryos expressing bovine TWIST2 at 35 hpf. (A) Representative images of zebrafish embryos injected with either a control construct (pmitfa_EGFP) or a construct driving the expression of bovine TWIST2 under control of the zebrafish *mitfa* promoter (pmitfa_btaTWIST2_EGFP). Melanocyte counts for head and trunk are given in brackets. Scale bars correspond to 1 mm. (B) Melanocytes in the head and trunk were counted in three experiments (controls: n = 14, 16, 19; TWIST2: n = 17, 16, 19). (C) The experiments were repeated with another set of constructs that additionally had CFP as a reporter for transgenesis. In transgenic animals, cerulean fluorescence in the eyes becomes visible at ~4–5 dpf. (D) Melanocyte counts in two replicate experiments at 35 hpf from zebrafish injected with the constructs pmitfa_EGFP_cryst_CFP and pmitfa_TWIST2_EGFP_cryst_CFP. If (B) and (D) are taken together the TWIST2 overexpressing embryos had significantly fewer melanocytes in the head in 4 out of 5 experiments and in the trunk in 3 out of 5 experiments.

We then quantified the number of visible melanocytes at 35 hpf in TWIST2 overexpressing versus control embryos in three independent experimental replicates (Fig 4A and 4B). Our strategy was expected to lead to mosaic animals, where only a fraction of the cells integrated the expression casettes into the genome. Furthermore, not all injected embryos were expected to become transgenic with this method. We therefore prepared a second pair of expression constructs, pmitfa_EGFP_cryst_CFP and pmitfa_btaTWIST2_EGFP_cryst_CFP, which additionally drive the expression of cerulean fluorescent protein (CFP) under control of the lens-specific gamma-crystallin promoter. Successful trangenesis with these constructs becomes visible at 5 dpf by the appearance of cerulean fluorescence in the eyes (Fig 4C). Using these constructs we injected two further sets of embryos and quantified the melanocytes at 35 hpf as before. These embryos were continued to grow after the melanocyte counting until 5 dpf. The embryos were then imaged again and embryos that were not transgenic were excluded from the analysis of melanocyte counts (Fig 4D).

Both sets of constructs showed comparable results. We analyzed three replicates with the first set of constructs and two replicates with the second set of constructs. In each of these five experiments melanocytes were counted separately in the head and in the trunk region. Injection with the TWIST2 overexpressing constructs led to a significant reduction in the mean number of head melanocytes in 4 out of the 5 replicate experiments. Trunk melanocytes were significantly reduced in 3 out of 5 replicate experiments. The same trend was visible in those experiments that did not show a significant difference.

## Discussion

In this study, we identified the amplification of a 6 kb genomic sequence in the 5’-flanking region of the *TWIST2* gene as most likely causative variant for the belted phenotype in cattle. The haplotype analysis refined the critical interval to only 37 kb. Although very precise, this mapping result has to be interpreted with some caution as it is dependent on a single recombinant chromosome that we observed in one Belted Galloway. A more conservative definition of the critical interval would have resulted in a ~290 kb interval (chr3:118,569,738–118,859,918), supported by >5 recombined chromosomes on either side. The small size of the shared haplotype is consistent with a relatively old origin of the *bt* allele, having arisen before the separation of modern cattle breeds. A 15^th^ century painting from Austria already depicts a belted ox suggesting that this allele is at least 600 years or roughly 200 generations old (S1 Fig). Historic records are in agreement with a central European origin of the *bt* allele and indicate that the *bt* allele may subsequently have spread north-west and been introgressed into Dutch cattle to eventually form the Dutch Belted cattle and into Scottish cattle to eventually form the Belted Galloways [26].

The precise fine-mapping facilitated the search for candidate causative variants. The 37 kb critical interval did not contain any gaps in the reference genome assembly. Thus, our Illumina re-sequencing approach had a good probability to really unravel all sequence variants in this relatively small region. There are no protein-coding nucleotides in the 37 kb critical interval. Even in the more conservative 290 kb interval, there are only 483 protein coding nucleotides encoding the entire 160 amino acid bovine TWIST2 protein. We can reliably exclude the possibility of any protein coding variants being the cause of the belted phenotype. The CNV was the best associated variant in the critical interval and showed the expected genotypes in 1653 out of 1655 genotyped cattle (99.9%). As the phenotype assignment in the two discrepant animals was solely based on their identity documents, we assume that in these two animals the phenotypes were incorrectly assigned (see Materials and methods). Other potential explanations for the imperfect association could be additional mutation events on the *bt*-associated haplotype that might have affected the ddPCR genotyping method (e.g. in one of the primer binding sites) or heterogeneity.

The strictly dominant mode of inheritance of the belt phenotype suggested a gain-of-function allele, such as e.g. an allele with a non-coding regulatory variant leading to ectopic expression of a gene. *TWIST2* represents an obvious candidate gene for such a potentially ectopically expressed gene. It is the only annotated protein-coding gene within 250 kb of the critical interval and the belt-associated CNV is actually located in the 5’-flanking region of *TWIST2*. TWIST2 is a basic helix loop helix (bHLH) transcription factor with a postulated role in early development influencing chondrogenic and dermal tissues [23,27]. Very recently another role for TWIST2 in the repair and regeneration of adult type IIb myofibers has been demonstrated [28]. *TWIST2* loss-of-function variants in humans cause several complex malformation disorders: ablepharon-macrostomia syndrome (AMS, MIM 200110), Barber-Say syndrome (BSS, MIM 209885) and Setleis syndrome or focal facial dermal dysplasia type III (FFDD3, MIM 227260), all being ectodermal dysplasias with complex facial malformations [29–31]. In one Setleis patient, hypo- and hyperpigmentation of the skin was reported [32]. Loss of *Twist2* function in mice leads to runting, cachexia (adipose and glycogen deficiency), and thin skin with sparse hair [33]. Additional findings are corneal thinning and a reduced population of stromal keratocytes, which are derived from the neural crest [34].

bHLH transcription factors are known to dimerize via their bHLH domain and each monomer binds to one halfsite of the DNA binding site. MITF is also a bHLH transcription factor absolutely required for the development of melanocytes. TWIST2 has been reported to heterodimerize with other bHLH transcription factors [23]. Thus, we suggest the following hypothetical mechanism for the expression of the belt phenotype: Ectopically expressed TWIST2 in the developing neural crest of belted cattle might interact in a dominant-negative manner with other bHLH transcription factors (MITF or its partners) and thus lead to either a reduction in the number of cells programmed for the melanoblast lineage or a reduced survival/proliferation of melanoblasts in the developing neural crest. Instead of this protein-protein interaction model with other bHLH transcription factors, ectopically expressed TWIST2 might also directly bind to DNA and repress the transcription of genes essential for melanoblast fate determination, such as e.g. *MITF*.

At this time, the proposed scenario is only a hypothesis and cannot be considered proven. It is currently not possible to directly investigate TWIST2 expression in the neural crest of bovine embryos. Our analysis of *TWIST2* RNA expression in adult skin from *bt/wt* heterozygous cattle clearly indicated that amplification of the CNV led to altered *TWIST2* expression. Thus, the data supported a regulatory effect of the CNV. However, in adult skin, we observed downregulated *TWIST2* mRNA expression from the *bt*-allele. Although our hypothesis postulates upregulation of *TWIST2*, we think that this observation does not completely refute the hypothesis as it still seems possible that the regulatory effect of the CNV might be directed in the opposite direction in other cell types and during other developmental stages, e.g. neural crest development. Our functional experiments in transgenic zebrafish support such a scenario, since overexpression of bovine TWIST2 led to a reduction in the number of neural-crest derived melanocytes. This observation, in conjunction with the pigmentary effects of mutant alleles in humans [32], is consistent with our proposal that mis-regulated expression of TWIST2 results in the belted pigmentation phenotype. A definitive test will require access to belted and non-belted bovine embryos.

Taken together, the genetic data strongly support a causal role of the CNV in the 5’-flanking region of the *TWIST2* gene for the belted phenotype in cattle. Our functional data further support the hypothesis of a regulatory variant that leads to altered expression of TWIST2. The belt is most likely the result of a decrease in the number of melanoblasts/melanocytes at an early stage in the developing embryo. In common with other mammals, the midsection of the trunk is particularly sensitive to such a decrease. Belted cattle thus probably represent a spontaneous mutant, in which a structural variant led to subtle changes in gene expression and consequently to an appealing coat color phenotype that was actively selected by cattle breeders. This study highlights the scientific value of spontaneous mutants, which have arisen during animal domestication. Billions of domestic animals have been handled and observed by their human owners providing us with all kinds of fascinating heritable phenotypes. Given the essential role of TWIST2 in development and the dramatic consequences of *TWIST2* coding variants, it is not surprising that the specific mechanism leading to the bovine belt phenotype so far has only been observed once in the entire history of animal domestication.

## Materials and methods

### Ethics statement

All animal experiments were performed according to the local regulations. The collection of cattle samples was approved by the ‘‘Cantonal Committee For Animal Experiments” (Canton of Bern; permits BE77/13 and BE75/16). Zebrafish were raised at the Institute of Anatomy, University of Bern (animal house licence number BE413 and animal experimentation number BE15/95). Zebrafish experiments at the University of Bath were reviewed by the Animal Welfare and Ethical Review Body (AWERB), and covered under the Home Office Project License PPL30/2937.

### Cattle samples

We isolated genomic DNA from hair roots or EDTA blood samples from 112 belted animals (4 belted Brown Swiss, 68 Belted Galloway, 40 Lakenvelder) and 267 non-belted controls from different breeds for SNP chip genotyping and the fine-mapping experiment. For the subsequent association study we recruited more than 1,000 additional DNA samples from the routine parentage testing performed at the University of Göttingen. The phenotype of the additional animals was solely based upon owners’ reports. During the investigation we excluded several animals that were reported as belted by the owners, but had only minimal white spots that did not resemble the typical belt phenotype. We also excluded several animals that were reported as non-belted by the owners, but turned out to be White Galloways, on which a belt would not have been visible [35]. The final cohort thus consisted of 333 belted animals and 1,322 non-belted cattle. In this cohort, we had 2 supposedly belted animals with discordant genotypes (Table 1). We were not able to verify the phenotypes of these two questionable animals as either the owners could not be reached or the animals had been slaughtered several years before the investigation and no photos or other information was available.

### SNP chip genotyping and haplotype analyses

Genotyping for the fine-mapping experiment was done on Illumina bovine HD beadchips containing 777,962 SNPs by GeneSeek/Neogen. Genotypes were stored in a BC/Gene database version 3.5 (BC/Platforms) and phased with SHAPEIT [36]. The haplotypes for chromosome 3 were exported into an Excel-file and visually inspected for shared segments among the 112 cases.

### Whole genome sequencing

We prepared a fragment library with approximately 300 bp insert size from a homozygous belted Brown Swiss cow (GU21) and a homozygous Belted Galloway bull (BG11) and collected 2x100 bp paired-end reads on an illumina HiSeq2000 instrument. We obtained 16.6x coverage on GU21 and 8.6x coverage on BG011. We mapped the reads to the UMD 3.1 reference genome with the Burrows-Wheeler Aligner (BWA) version 0.5.9-r16 with default settings [37]. After sorting the mapped reads by the coordinates of the sequence with Picard tools, we labeled the PCR duplicates also with Picard tools (http://sourceforge.net/projects/picard/). We used the Genome Analysis Tool Kit (GATK version 0591) to perform local realignment and to produce a cleaned BAM file [38]. Variant calls were then made with the Unified Genotyper module of GATK. For variant calling we used only reads with mapping quality of ≥ 30 and bases with quality values ≥ 20. The variant data output file obtained in VCF format 4.0 was filtered for high quality SNPs using the variant filtering module of GATK. The hard filtering of variants was done as explained in the GATK best practice manual. The snpEFF software [39] together with the UMD 3.1 annotation (ENSEMBL release 79) was used to predict the functional effects of detected variants.

### Droplet digital PCR (ddPCR)

To determine the copy number of the CNV on chromosome 3 we designed a ddPCR assay. Primers and probes were designed using the Primer3 program (http://bioinfo.ut.ee/primer3-0.4.0/primer3/). An 86 bp amplicon spanning chr3:118,612,280–118,612,365 was used (fwd GATGAGTGTTCTGGGTGGAAG; rev CTGTGTCTGCCCATCTCTG; probe FAM- AGGTCCCTAGTCTCTGCCTTCCC-BHQ1) to detect the CNV. A 96 bp fragment of the coagulation factor II thrombin (F2) gene (chr15:77,533,575–77,533,670) served as control amplicon. This genomic region showed equal copy-numbers among the sequenced cattle genomes and has no known role in coat color genetics. The sequences of the primers and probe for the *F2* gene were: fwd CCTGTCTGCTGAGACGCCG; rev GTGGTAGAGTTGATTCTGGAATAGAAAGCAT; probe HEX- CCCCGCCACCCGCAGTGTCT-BHQ1. Between 50–150 ng of genomic DNA were digested using 10 units of *Bam*HI restriction enzyme. The CNV has a *Bam*HI restriction site at position chr3:118,612,241, close to the ddPCR amplicon. The restriction digest was prepared in 1x ddPCR Supermix for Probes (Bio-Rad). After incubation at 37°C for 60 min, primers (900 nM each) and probes (250 nM each) were added directly to the mix and droplets were generated using the QX200 Droplet Generator (Bio-Rad). The reactions were amplified with the following temperature profile: initial denaturation for 10 min at 95°C, followed by 40 cycles of 30 s at 95°C and 60 s at 55°C, and a final droplet stabilization at 98°C for 10 min. Droplets were analyzed with the QX200 Droplet Reader and the software provided by the manufacturer (Bio-Rad).

### Inverse PCR for the characterization of CNV breakpoints

Primers CNV_R1, GAAGCGTGGTCCTGTGAAGA, and CNV_F1, AGGGGGAAGGCAGAGACTAG, in combination with the GoTaq long range polymerase (Promega) were used to amplify across the breakpoints of the amplified region in belted cattle. PCR conditions were 60 s initial denaturation at 94°C, followed by 30 cycles of 30 s at 95°C, 4 min at 60°C, and 10 min at 66°C. The resulting 4,845 bp product was sequenced by the Sanger method with the PCR primers and a set of internal primers on an ABI3730 capillary sequencing instrument (Thermo Fisher Scientific).

### RNA-seq

We collected 6 mm skin biopsies, in the dorsal pigmented region, ~5 cm caudal of the belt, from 4 adult belted cows (BG155, GU448, GU466, GU469) and 3 adult non-belted cows (GU449, GU467, GU468) and isolated total RNA. The TruSeq stranded mRNA library prep kit (Illumina) was used to prepare libraries, which were sequenced on the Illumina HiSeq 3000 platform using 2 x 150 bp paired-end sequencing cycles. The reads in FASTQ format were processed for quality checks using the FastQC tool (v0.10.1; http://www.bioinformatics.babraham.ac.uk). Good quality reads were then mapped to the cattle reference genome UMD 3.1 using the STAR Aligner [40]. We allowed for a maximum of four mismatches per read pair and a maximum intron length of 100 kb. We used htseq-count from the HTSeq package to count reads against annotated Ensembl genes models (version 79) Differential expression was carried out on raw read counts for genes using edgeR [41]. For the second experiment we used 7 RNA samples from 4 different heterozygous *bt/wt* animals, which were also heterozygous A/G at UMD3.1:chr3:118,689,836A>G in the 3’-UTR of the *TWIST2* gene (GU456_skin_pigmented, GU456_skin_unpigmented, GU460_skin_pigmented, GU460_skin_unpigmented, GU462_skin_pigmented, GU462_skin_unpigmented, GU466_skin_pigmented). As controls we used skin RNA samples from one adult non-belted cow (GU467) and previously published data of 7 RNA samples derived from fetal skin taken from the head region of bovine fetuses [42]. The 8 non-belted animals were also heterozygous A/G at UMD3.1:chr3:118,689,836A>G to allow the differentiation of the alleles. Relative quantification was achieved by counting the RNA-seq reads in each of the 15 samples for the A- and the G-allele at UMD3.1:chr3:118,689,836A>G. We obtained whole genome sequencing data from the animal GU466 using the 10xGenomics technology at ~30x coverage. Haplotype-phasing using the long-ranger software (10xGenomics) established that the *bt*-associated haplotype contained the G-allele at UMD3.1:chr3:118,689,836A>G.

### Recombinant constructs for the expression of bovine TWIST2

An 4.1 kb *mitfa* promoter fragment, the coding sequences for EGFP and bovine TWIST2, and the IRES casette were amplified separately and engineered into a vector containing Tol2 sites (pTKXIGΔin; Kawakami Laboratory) using the Gibson Assembly^®^ Cloning kit from NEB. We thus generated the plasmids pmitfa_EGFP (control construct without TWIST2 expression cassette) and pmitfa_btaTWIST2_EGFP. A detailed map of these plasmids is given in S2 and S3 Figs.

An expression casette containing the gamma-crystallin promoter and the coding sequence of CFP was taken from *Tg(hsp70l*::*caALK5)* [43] and inserted into pmitfa_EGFP and and pmitfa_btaTWIST2_EGFP by Gibson cloning to generate pmitfa_EGFP_cryst_CFP and pmitfa_TWIST2_EGFP_cryst_CFP. A detailed map of these plasmids is given in S4 and S5 Figs. Plasmid DNA was purified using the Qiagen HiSpeed Maxiprep kit and the correct sequence was verified by Sanger sequencing.

### Zebrafish embryo microinjections

Injections were performed on embryos of wildtype strains AB and Tg(myl7:GFP) [44] at the one cell stage. Approximately 2 to 3 nl of injection mixture was injected in each embryo using a FemtoJet Microinjector (Eppendorf). Injection mixture comprised 1 μl of construct DNA at 100 ng/μl concentration, 1 μl of Tol2 transposase mRNA at 50 ng/μl and 1 μl of phenol red for identification of injected embryos. Six to eight hours after the injections embryos were sorted and unfertilized or damaged embryos were discarded.

### Melanocyte imaging and counting

At approximately 24 hpf, the dishes were cleaned and the medium was changed. The embryos were grown at 28°C and screened at about 35 hpf. Both the controls and TWIST2 expressing embryos were carefully dechorionated, put in tricaine treated fish water (final tricaine concentration 160 mg/l) and used for melanocyte counting. For counting purposes, the embryos were imaged using bright field technique under an SMZ25 fluorescent stereoscope (Nikon). The embryos were imaged lying laterally with their heads facing to the right using methyl cellulose to prevent them from drying. The images were coded and melanocytes were counted by an investigator who was blinded to the information whether embryos were injected with control or TWIST2 overexpressing constructs.

In the experiments with the constructs pmitfa_EGFP_cryst_Cer and pmitfa_TWIST2_EGFP_cryst_Cer, embryos were further grown in single well dishes until 5 dpf. At this time they were imaged under an SMZ25 fluorescent stereoscope (Nikon) and screened for presence or absence of cerulean fluorescence in the eyes. Melanocyte counts from 35 hpf were only used for the analysis, if the embryos showed positive cerulean fluorescence in the eyes at 5 dpf.

### Antibody labelling and fluorescent microscopy

After counting melanocytes, the embryos were fixed with 2% PFA diluted in PBS, overnight at 4°C and were processed for antibody labelling as described previously [45]. The anti-GFP antibody was obtained from Aves Labs Inc., the anti-Sox10 antibody was obtained from Gene tex. Anti-GFP and anti-Sox10 were used in a dilution of 1:500. Secondary antibodies were anti-chicken coupled to Alexa Fluor^®^ 568 (1:500; ThermoScientific) and anti-rabbit coupled to Alexa Fluor^®^ 647 (1:250; Invitrogen). Nuclei were counterstained with 4c,6-diamidino-2-phenylindole dihydrochlorid (DAPI; Invitrogen). After antibody labelling, the embryos were imaged under an AxioLSM880 confocal microscope (Zeiss). Whole mount embryos were imaged under 10x, the images were processed using ImageJ and Adobe Illustrator.

### Sequence data accession numbers

Whole genome sequencing data and RNA-seq data from this study have been submitted to the European Nucleotide Archive under accessions PRJEB14604 and PRJEB14606.

## Supporting information

S1 Fig Historical evidence for the origin of the belted phenotype. **(A)** Painting “Birth of Jesus Christ” from the “Albrechts’s altar” at Stift Klosterneuburg in Austria. The painting is dated to 1438/1439 and shows an ox with the belted phenotype. This suggests that the belted phenotype is more than 500 years old and may have originated in alpine cattle before the strict separation of the modern cattle breeds. Photo: Michael Himml, Vienna. **(B)** Pencil drawing “The Lakenvelder” by the Dutch artist François Ryckhals (1609–1647), which is exhibited at the Niedersächsische Landesmuseen Braunschweig in Germany. The Lakenvelder (Dutch Belted) breed traces directly to the original belted cattle, which were described in Appenzell Switzerland and Austria. The breed was then established in the Netherlands in the 17^th^ century [26].
(PDF)

S2 Fig Map of expression plasmid pmitfa_EGFP. This plasmid drives the expression of EGFP under the control of the zebrafish *mitfa* promoter. The correct cloning of the functional elements was verified by Sanger sequencing.
(PDF)

S3 Fig Map of expression plasmid pmitfa_btaTWIST2_EGFP. Map of the expression plasmid pmitfa_btaTWIST2_EGFP. This plasmid drives the expression of bovine TWIST2 under the control of the zebrafish *mitfa* promoter. The correct cloning of the functional elements was verified by Sanger sequencing. Expression of EGFP probably does not work in zebrafish as the IRES is not reliably functioning in this species.
(PDF)

S4 Fig Map of expression plasmid pmitfa_EGFP_cryst_CFP. This plasmid drives the expression of EGFP under the control of the zebrafish *mitfa* promoter. In a second expression cassette, the open reading frame of cerulean fluorescent protein (CFP) is driven under the control of the lens-specific gamma-crystallin promoter. The correct cloning of the functional elements was verified by Sanger sequencing.
(PDF)

S5 Fig Map of expression plasmid pmitfa_TWIST2_EGFP_cryst_CFP. This plasmid drives the expression of bovine TWIST2 under the control of the zebrafish *mitfa* promoter. In a second expression cassette, the open reading frame of cerulean fluorescent protein (CFP) is driven under the control of the lens-specific gamma-crystallin promoter. The correct cloning of the functional elements was verified by Sanger sequencing. Expression of EGFP probably does not work in zebrafish as the IRES is not reliably functioning in this species.
(PDF)

S1 Table Fine-mapping of the belt locus. (XLSX)

S2 Table Detailed CNV copy numbers as determined by ddPCR. (XLSX)

S3 Table Repeat content of the CNV. (XLSX)

S4 Table RNA-seq experiments. (XLSX)
