# Supplementary figures and images for: A structural variant in the 5’-flanking region of the TWIST2 gene affects melanocyte development in belted cattle
Source: PLoS One. 2017 Jun 28;12(6):e0180170. doi: 10.1371/journal.pone.0180170 (PMC5489250; doi:10.1371/journal.pone.0180170)

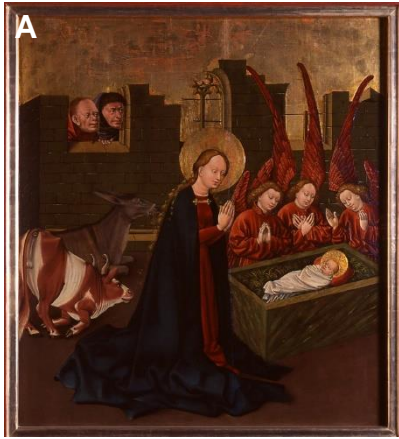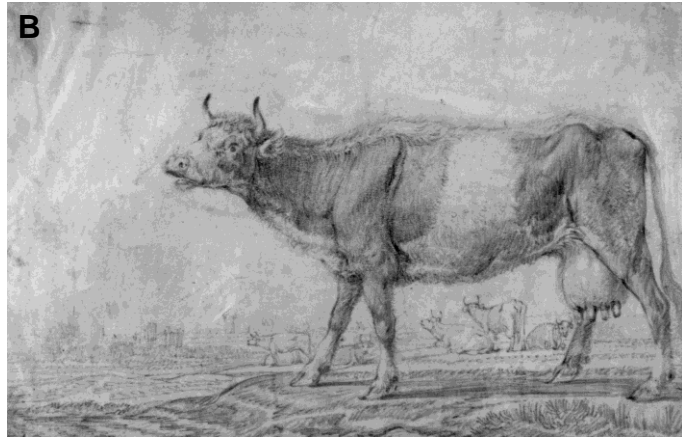

Supplement: S1 Fig — (A) Painting “Birth of Jesus Christ” from the “Albrechts’s altar” at Stift Klosterneuburg in Austria. The painting is dated to 1438/1439 and shows an ox with the belted phenotype. This suggests that the belted phenotype is more than 500 years old and may have originated in alpine cattle before the strict separation of the modern cattle breeds. Photo: Michael Himml, Vienna. (B) Pencil drawing “The Lakenvelder” by the Dutch artist François Ryckhals (1609–1647), which is exhibited at the Niedersächsische Landesmuseen Braunschweig in Germany. The Lakenvelder (Dutch Belted) breed traces directly to the original belted cattle, which were described in Appenzell Switzerland and Austria. The breed was then established in the Netherlands in the 17th century [26]. (PDF) [file pone.0180170.s001.pdf]

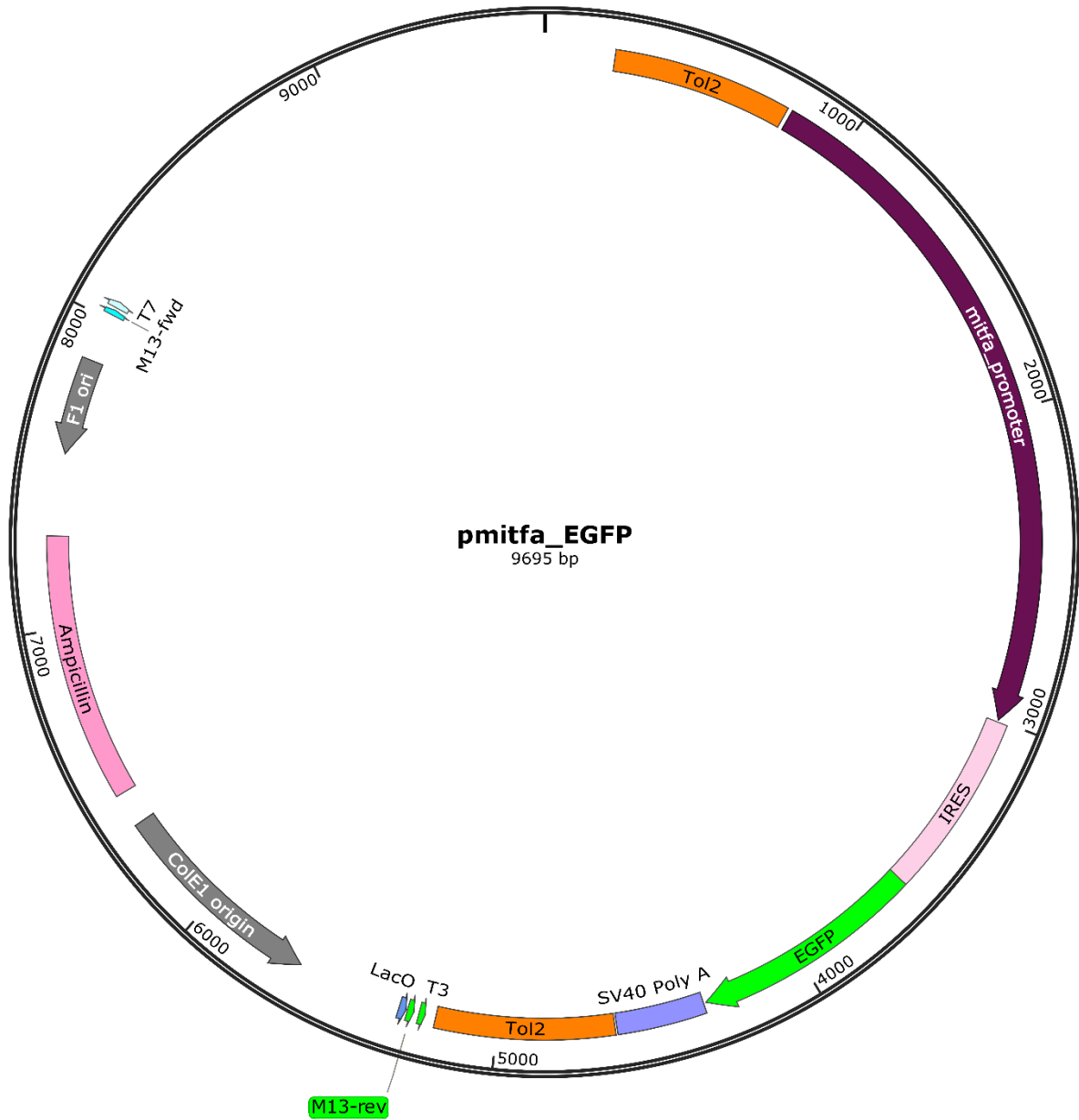

Supplement: S2 Fig — This plasmid drives the expression of EGFP under the control of the zebrafish mitfa promoter. The correct cloning of the functional elements was verified by Sanger sequencing. (PDF) [file pone.0180170.s002.pdf]

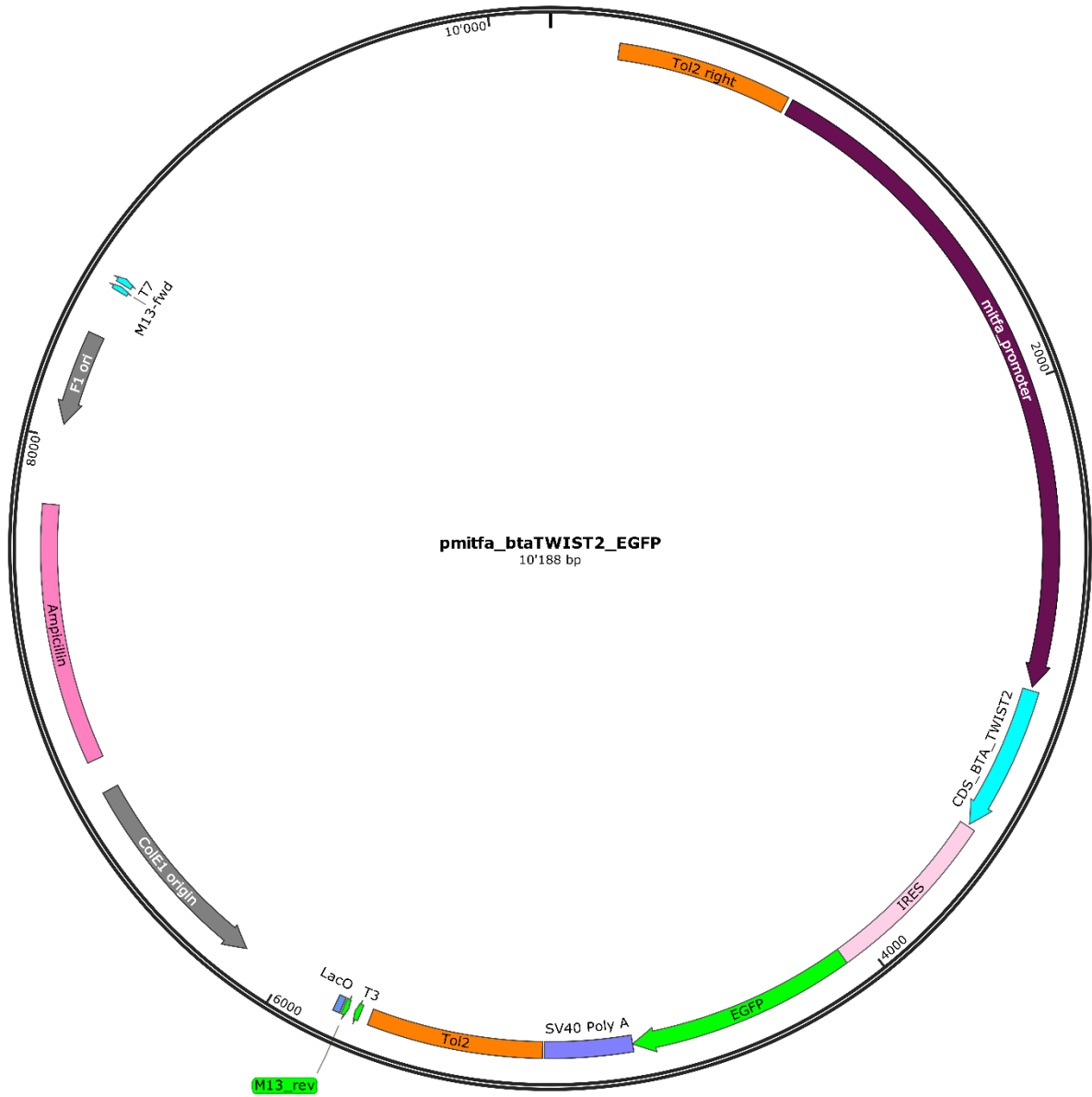

Supplement: S3 Fig — Map of the expression plasmid pmitfa_btaTWIST2_EGFP. This plasmid drives the expression of bovine TWIST2 under the control of the zebrafish mitfa promoter. The correct cloning of the functional elements was verified by Sanger sequencing. Expression of EGFP probably does not work in zebrafish as the IRES is not reliably functioning in this species. (PDF) [file pone.0180170.s003.pdf]

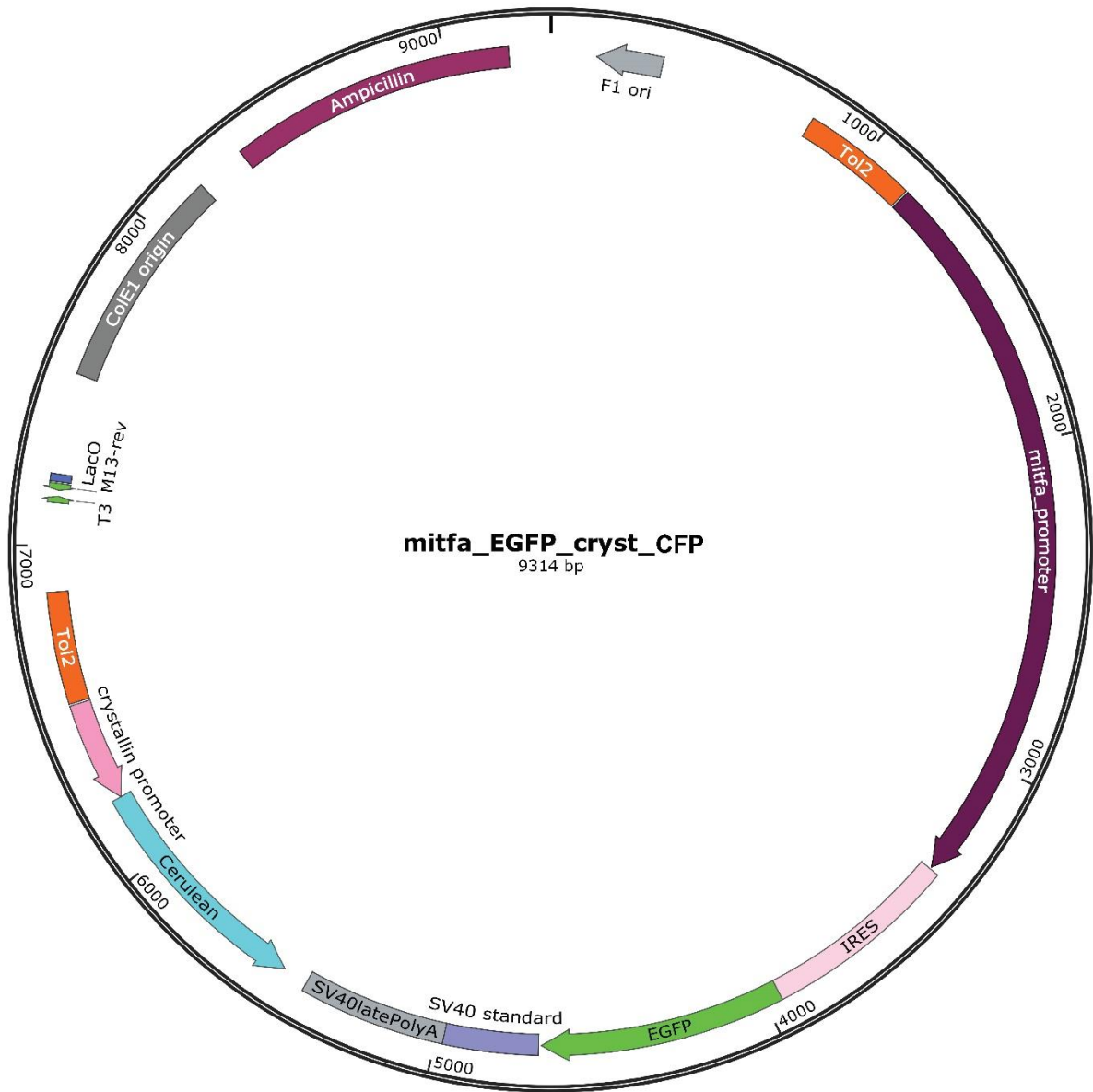

Supplement: S4 Fig — This plasmid drives the expression of EGFP under the control of the zebrafish mitfa promoter. In a second expression cassette, the open reading frame of cerulean fluorescent protein (CFP) is driven under the control of the lens-specific gamma-crystallin promoter. The correct cloning of the functional elements was verified by Sanger sequencing. (PDF) [file pone.0180170.s004.pdf]

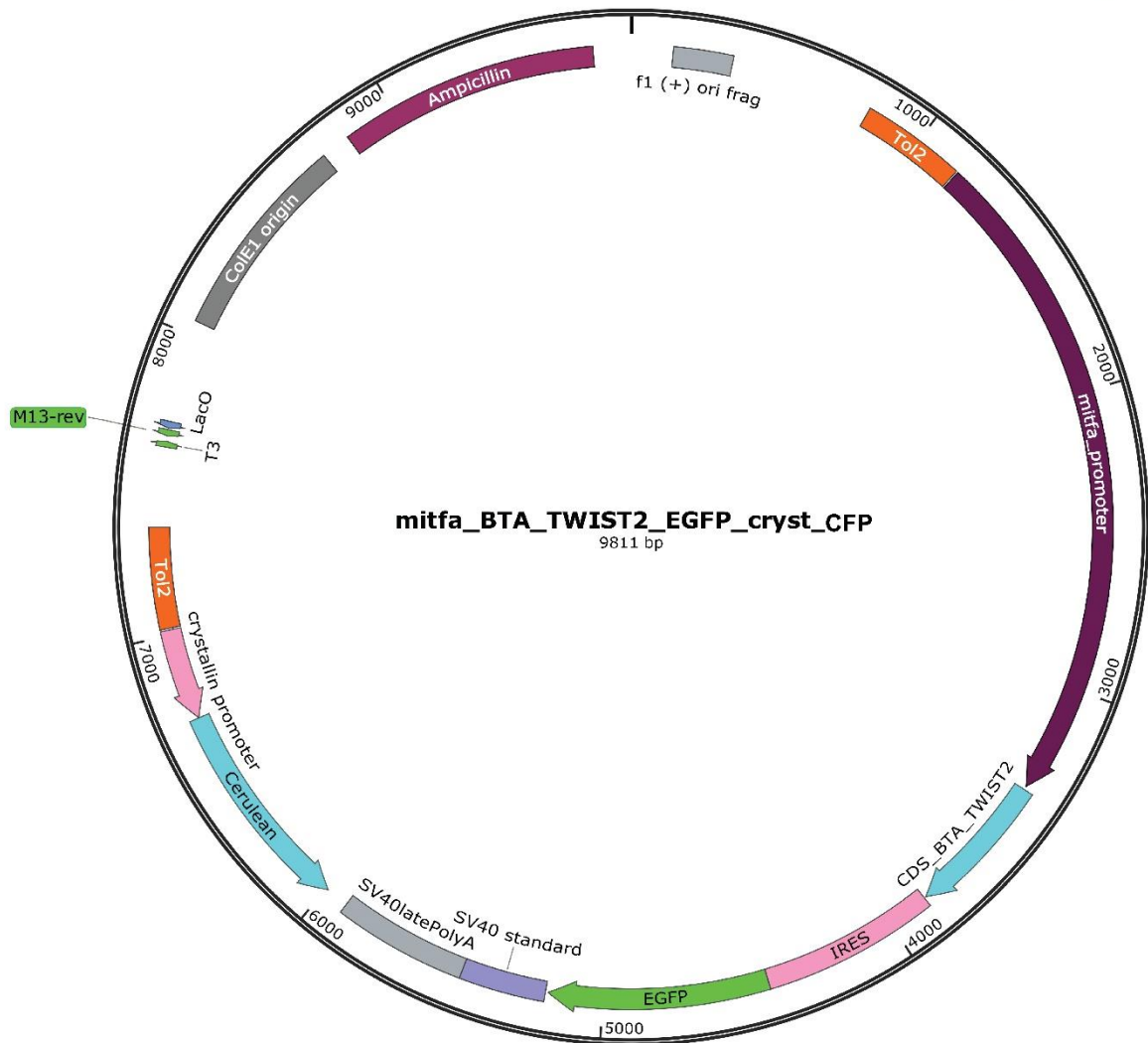

Supplement: S5 Fig — This plasmid drives the expression of bovine TWIST2 under the control of the zebrafish mitfa promoter. In a second expression cassette, the open reading frame of cerulean fluorescent protein (CFP) is driven under the control of the lens-specific gamma-crystallin promoter. The correct cloning of the functional elements was verified by Sanger sequencing. Expression of EGFP probably does not work in zebrafish as the IRES is not reliably functioning in this species. (PDF) [file pone.0180170.s005.pdf]
